# Broussonin A– and B–mediated inhibition of angiogenesis by blockade of VEGFR‐2 signalling pathways and integrin β1 expression

**DOI:** 10.1111/jcmm.17173

**Published:** 2022-01-06

**Authors:** Jae Hyeon Kim, Sunho Kim, Surim Han, Eun‐Kyung Ahn, Young‐Rak Cho, Wonsik Jeong, Sung Joon Kim, Gyu‐Un Bae, Joa Sub Oh, Dong‐Wan Seo

**Affiliations:** ^1^ Department of Pharmacy College of Pharmacy Dankook University Cheonan Republic of Korea; ^2^ Biocenter Gyeonggi Business & Science Accelerator Suwon Republic of Korea; ^3^ Department of Pharmacy College of Pharmacy Sookmyung Women’s University Seoul Republic of Korea

**Keywords:** broussonin A, broussonin B, integrin β1, vascular endothelial growth factor

## Abstract

In the present study, we demonstrate the regulatory effects and mechanism of broussonin A and B, diphenylpropane derivatives isolated from *Broussonetia kazinoki*, on vascular endothelial growth factor‐A (VEGF‐A)–stimulated endothelial cell responses in vitro and microvessel sprouting ex vivo. Treatment with broussonin A or B suppressed VEGF‐A‐stimulated endothelial cell proliferation by regulating the expression of cell cycle–related proteins and the phosphorylation status of retinoblastoma protein. In addition, treatment with broussonin A or B abrogated VEGF‐A‐stimulated angiogenic responses including endothelial cell migration, invasion, tube formation and microvessel formation from rat aortic rings. These anti‐angiogenic activities of broussonin A and B were mediated through inactivation of VEGF‐A‐stimulated downstream signalling pathways, localization of vascular endothelial‐cadherin at cell‐cell contacts, and down‐regulation of integrin β1 and integrin‐liked kinase. Furthermore, treatment with broussonin A or B inhibited proliferation and invasion of non–small cell lung cancer and ovarian cancer cells. Taken together, our findings suggest the pharmacological potential of broussonin A and B in the regulation of angiogenesis, cancer cell growth and progression.

## 1 INTRODUCTION

Angiogenesis, the sprouting of endothelium‐lined vascular structures, contributes to the pathophysiology of diseases including cancer, diabetic retinopathy and ischaemic vascular diseases. Vascular endothelial growth factor (VEGF) has long been appreciated as a key therapeutic target for the treatment of diseases associated with pathological angiogenesis.^1^, ^2^ However, most drugs targeting VEGF and VEGF‐mediated signalling pathways in clinical use often lead to disease recurrence and progression associated with drug resistance. Recent studies demonstrate that VEGF within the tumour microenvironment targets many different types of cells including immune cells, fibroblasts and tumour cells as well as endothelial cells.^3^ Furthermore, VEGF‐mediated signalling is complicatedly regulated by interactions with VEGF receptors (VEGFRs) and other cell signalling‐related proteins including neuropilins, integrins and proteoglycans.^3^, ^4^

These complex dynamics of VEGF‐mediated signalling networks may partially explain the limitation of anti‐VEGF/VEGFR therapy in the clinic. Therefore, in‐depth investigations of integrative signalling pathways within the tumour microenvironment may provide crucial therapeutic targets and strategies for improving clinical outcomes.

*Broussonetia kazinoki* (*B*. *kazinoki*) Siebold (Moraceae), a deciduous shrub distributed in Eastern Asia including Korea, China and Japan, has been traditionally used as a folk medicine for the treatment of amblyopia, inflammation and oedema as well as a raw material for making high‐quality paper in Korea. The extract and bioactive components isolated from *B*. *kazinoki* have been reported to possess various pharmacological properties including myogenic, anti‐allergic, anti‐inflammatory, anti‐diabetic and anti‐tumour activities.^5^, ^6^, ^7^, ^8^, ^9^, ^10^, ^11^ We have previously demonstrated that the ethanolic extract of *B*. *kazinoki* or marmesin, a furanocoumarin component isolated from *B*. *kazinoki*, negatively modulates VEGF‐A‐induced angiogenic responses by inactivation of VEGF‐A/VEGFR‐2‐mediated signalling network.^12^, ^13^ Furthermore, marmesin exerts anti‐proliferative and anti‐invasive activities against non–small cell lung cancer (NSCLC) cells through the inhibition of mitogen‐induced signalling pathways.^14^ Marmesin‐mediated suppression of VEGF expression and secretion from NSCLC cells is associated with modulation of tumour angiogenesis.^14^

Broussonin A (2‐[3‐(4‐hydroxyphenyl)propyl]‐5‐methoxyphenol), a diphenylpropane derivative isolated from several plants including *B*. *kazinoki*, *B*. *papyrifera* and *Anemarrhena asphodeloides* (*A*. *asphodeloides*), has been reported to possess antiviral, anti‐inflammatory, anti‐adipogenic and oestrogenic properties.^15^, ^16^, ^17^, ^18^ In addition, broussonin B (4‐[3‐(4‐hydroxyphenyl)propyl]‐3‐methoxyphenol) isolated from *B*. *kazinoki* and *A*. *asphodeloides* has been known to exert anti‐adipogenic and neurotrophic activities.^17^, ^19^ However, the effects and molecular mechanisms of broussonin A and B on angiogenesis which is closely associated with pathological conditions have never been elucidated. Therefore, the current study aims to determine the effects and action mechanisms of broussonin A and B isolated from edible branches of *B*. *kazinoki* on endothelial cell and cancer cell responses.

## 2 MATERIALS AND METHODS

### 2.1 Cell culture conditions

Human umbilical vein endothelial cells (HUVECs) from Lonza (Walkersville, MD, USA) were grown in EGM‐2^®^ BulletKit media and used between passages 4 and 6 for all experiments, according to the manufacturer's instructions (Lonza). Human non–small cell lung cancer (A549 and H1299) and ovarian cancer (SKOV‐3) cells from the American Type Culture Collection (Manassas, VA, USA) were cultured in 10% foetal bovine serum‐Dulbecco's modified Eagle's medium (FBS‐DMEM, Hyclone Laboratories, Logan, UT, USA).

### 2.2 Isolation and spectrometric analysis of broussonin A and B

**FIGURE 1:**
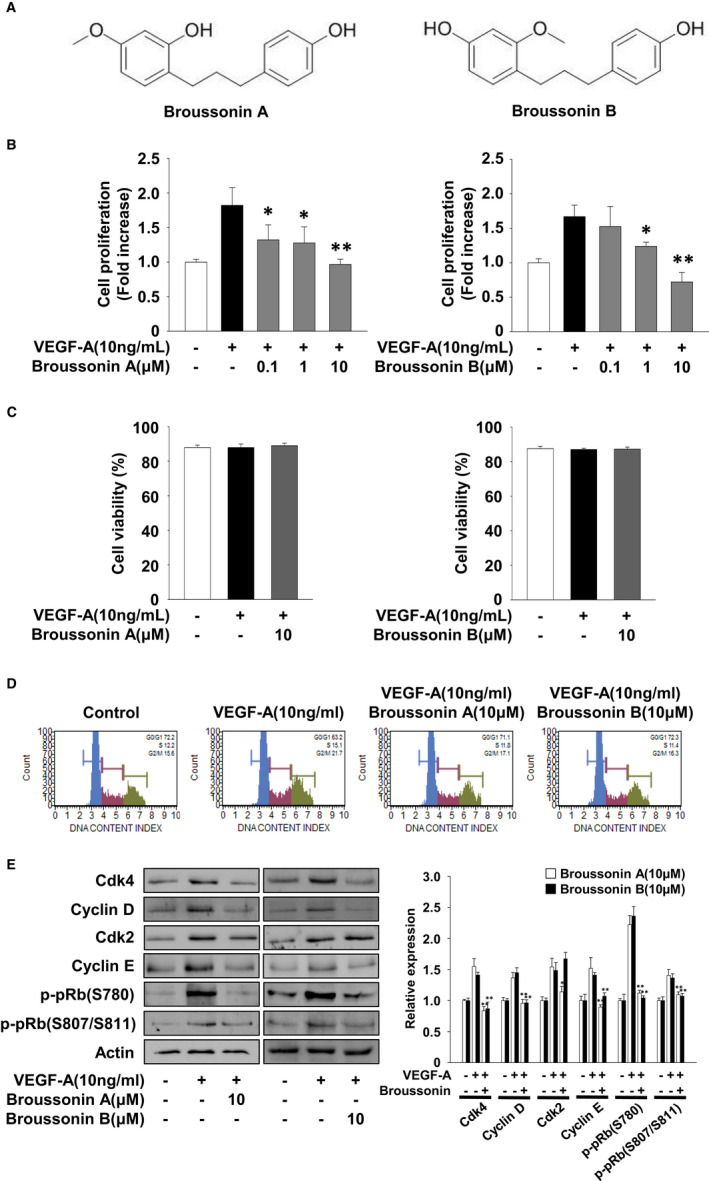
Broussonin A and B exert anti‐proliferative activity in VEGF‐A‐treated HUVECs. (A) The chemical structures of broussonin A and B. (B) Cell proliferation, (C) viability, (D) cell cycle and (D) Western blot analyses were performed as described in Materials and methods. Quiescent cells were pretreated with broussonin A or B (0.1–10 µM) for 30 min, followed by VEGF‐A (10 ng/mL) stimulation for 24 h. The results from three independent experiments (mean ± SD) are presented as (B) the fold‐increase of untreated controls or (C) the percentage of viable cells of total cell counts. (D, E) Results shown are representative of at least three independent experiments. Integrated density values were normalized to untreated controls. Statistical significance is indicated (*p < .05, **p < .01, compared with VEGF‐A‐treated cells)

Broussonin A and B were isolated in an ethyl acetate fraction partitioned from the ethanolic extract of *B*. *kazinoki*. High‐performance liquid chromatography (HPLC) analysis was performed on an Agilent 1200 series (Agilent Technologies, Santa Clara, CA, USA) using Kromasil^®^ C18 column (250 × 4.6 mm I.D., 5 µm particle size) (AkzoNobel, Bohus, Sweden) with a stepwise gradient elution of methanol‐0.05% trifluoroacetic acid in water (20%–100% methanol) at a flow rate of 1 mL/min. The purity of broussonin A and B by HPLC analysis was >98%. ^1^H‐ and ^13^C‐nuclear magnetic resonance (NMR) spectra of broussonin A and B were recorded on a Bruker Ascend 700 MHz NMR spectrometer (Bruker, Billerica, MA, USA). Broussonin A: ^1^H‐NMR (700 MHz, CD_3_OD_3_) 7.00 (2H, d, *J* = 8.4 Hz, H‐2'’, 6'’), 6.92 (1H, d, *J* = 8.4 Hz, H‐6’), δ 6.69 (2H, d, *J* = 8.4 Hz, H‐3'’, 5'’), 6.36 (1H, d, *J* = 2.1 Hz, H‐3’), 6.34 (1H, dd, *J* = 8.0, 2.1 Hz, H‐5’), 3.72 (3H, s, OCH_3_), 2.53 (4H, m, H‐1, 3), 1.81 (2H, m, H‐2); ^13^C‐NMR (175 MHz, CD_3_OD_3_) δ 158.9 (C‐4’), 155.6 (C‐2’), 154.8 (C‐4'’), 133.5 (C‐1'’), 129.7 (C‐6’), 128.9 (C‐2'’, 6'’), 121.0 (C‐1’), 114.6 (C‐3'’, 5'’), 104.0 (C‐5’), 100.9 (C‐3’), 54.1 (OCH_3_), 34.5 (C‐3), 32.1 (C‐2), 28.9 (C‐1); ESI(electrospray ionization)‐MS (mass spectrometry) (positive mode) *m*/*z* 259 [M + H]^+^. Broussonin B: ^1^H‐NMR (700 MHz, CD_3_OD_3_) 6.98 (2H, d, *J* = 8.4 Hz, H‐2'’, 6'’), 6.88 (1H, d, *J* = 7.7 Hz, H‐6’), δ 6.69 (2H, d, *J* = 8.4 Hz, H‐3'’, 5'’), 6.39 (1H, d, *J* = 2.8 Hz, H‐3’), 6.30 (1H, dd, *J* = 8.0, 2.0 Hz, H‐5’), 3.74 (3H, s, OCH_3_), 2.50 (4H, m, H‐1, 3), 1.77 (2H, m, H‐2); ^13^C‐NMR (175 MHz, CD_3_OD_3_) δ 158.9 (C‐2’), 156.9 (C‐4’), 155.4 (C‐4'’), 134.1 (C‐1'’), 130.4 (C‐6’), 129.5 (C‐2'’, 6'’), 122.0 (C‐1’), 115.2 (C‐3'’, 5'’), 106.7 (C‐5’), 98.9 (C‐3’), 54.8 (OCH_3_), 35.1 (C‐3), 32.8 (C‐2), 29.5 (C‐1); ESI‐MS (positive mode) *m*/*z* 259 [M + H]^+^. The structures of broussonin A and B are presented in Figure 1A. The stock solution (10 mM) of broussonin A and B was dissolved in 100% dimethyl sulfoxide.

### 2.3 Reagents

The following agents were obtained from commercial sources: vascular endothelial growth factor‐A 165 (Merck Millipore, Billerica, MA, USA); anti‐phospho‐VEGFR‐2 (Y951) (Abcam, Cambridge, UK); anti‐phospho‐p70^S6K^ (T421/S424), anti‐phospho‐Akt (S473), anti‐phospho‐ERK (T202/Y204), anti‐phospho‐p38^MAPK^ (T180/Y182), anti‐phospho‐pRb (S780) and anti‐phospho‐pRb (S807/S811) (Cell Signaling, Beverly, MA, USA); anti‐phospho‐tyrosine (BD Biosciences, Bedford, MA, USA); anti‐VEGFR‐2, anti‐vascular endothelial (VE)‐cadherin, anti‐integrin β1, anti‐ILK, anti‐p70^S6K^, anti‐Akt, anti‐ERK, anti‐p38^MAPK^, anti‐Cdk2, anti‐Cdk4, anti‐cyclin D, anti‐cyclin E, anti‐actin antibodies and mouse and rabbit IgG‐horseradish peroxidase conjugates (Santa Cruz Biotechnology, Santa Cruz, CA, USA); Alexa Fluor 488–conjugated goat anti‐mouse IgG (Life Technologies, Grand Island, NY, USA).

### 2.4 Cell proliferation and viability assay

HUVECs, plated on 6‐well plates (1 × 10^5^ cells/well, SPL Life Sciences, Gyeonggi‐do, Republic of Korea) were serum‐starved for 14 h in endothelial cell basal medium‐2 (EBM‐2, Lonza) and pretreated with broussonin A or B (0.1–10 µM) for 30 min, followed by VEGF‐A (10 ng/mL) stimulation for 24 h. In some experiments, human non–small cell lung cancer (A549 and H1299) and ovarian cancer (SKOV‐3) cells, plated on 6‐well plates (5 × 10^4^ cells/well), were serum‐starved for 24 h in basal DMEM and pretreated with broussonin A or B (10 µM) for 30 min, followed by 10% FBS stimulation for 24 h. Cell proliferation and viability were determined as described previously.^20^, ^21^, ^22^ Results from triplicate determinations (mean ± standard deviation) are presented as the fold‐increase of the untreated controls or the percentage of viable cells of total cell count.

### 2.5 Cell cycle analysis

Quiescent HUVECs were pretreated with broussonin A or B (10 µM) for 30 min, followed by VEGF‐A (10 ng/mL) stimulation for 24 h. Cells were fixed with ice‐cold 70% ethanol, stained with Muse™ cell cycle reagent, and then analysed by a Muse^TM^ cell analyser (Merck Millipore).

### 2.6 RNA purification and reverse transcriptase‐polymerase chain reaction (RT‐PCR)

Total RNA was isolated using PureHelix^TM^ Total RNA Purification kit (Nanohelix Co., Daejeon, Republic of Korea). RNA purity and concentration were determined using a NanoDrop^TM^ 2000 spectrophotometer (Thermo Fisher Scientific, Waltham, MA). One microgram of RNA was used as template for RT‐mediated PCR using 1^st^ Strand cDNA Synthesis kit (BioAssay Co., Daejeon, Republic of Korea). Primer sets for integrin β1 were forward 5’‐GAAGGGTTGCCCTCCAGA‐3’ and reverse 5’‐GCTTGAGCTTCTCTGCTGTT‐3’; and the primer sets for glyceraldehydes‐3‐phosphate dehydrogenase (GAPDH) were forward 5’‐GAAGGTGAAGGTCGGAGTC‐3’ and reverse 5’‐GAAGATGGTGATGGGATTTC‐3’.

### 2.7 Immunoprecipitation and Western blot analysis

Quiescent HUVECs were pretreated with broussonin A or B (10 µM) for 30 min, followed by VEGF‐A (10 ng/mL) stimulation for the indicated time points. Cells were lysed by incubation in 50 mM Tris‐HCl (pH 7.4), 1% Triton X‐100, 150 mM NaCl, 0.5 μg/mL leupeptin, 1 μg/mL pepstatin A, 10 μg/mL aprotinin, 100 μg/mL 4‐(2‐aminoethyl)benzenesulfonyl fluoride, 1 mM EDTA, 1 mM sodium orthovanadate, 25 mM sodium fluoride, 80 mM β‐glycerophosphate and 10% glycerol for 30 min at 4°C. Cell lysates were subjected to immunoprecipitation and Western blot as previously described.^23^, ^24^ Bands of interest were integrated and quantified by the use of National Institutes of Health (NIH) ImageJ version 1.51j8 software.

### 2.8 Cell migration assay

A single wound was created in the centre of confluent HUVEC monolayer by a sterile pipette tip. After serum starvation for 2 h, cells were pretreated with broussonin A or B (0.1–10 µM) for 30 min, followed by VEGF‐A (10 ng/mL) stimulation for 16 h. Following fixation with methanol, cells were stained with 0.04% Giemsa solution (Sigma‐Aldrich Co., St. Louis, MO, USA). The migration of cells across a wound field gap was quantified as previously described.^25^

### 2.9 Cell invasion assay

Transwell invasion assay was performed as previously described.^13^, ^26^, ^27^ HUVECs or cancer cells, plated on Matrigel^®^ (BD Biosciences)‐coated transwell inserts (Costar, 6.5 mm diameter insert, 8 μm pore size) (Corning Inc., Corning, NY, USA) were serum‐starved for 2 h and pretreated with broussonin A or B (1–10 μM) for 30 min, followed by VEGF‐A (10 ng/mL) or 10% FBS stimulation for 16 h. After fixation with methanol, invasive cells were stained with 0.04% Giemsa solution and quantified from six different fields using x200 objective magnification.

### 2.10 Immunofluorescence microscopy

Quiescent HUVECs, plated on gelatin‐coated coverslips in 12‐well plates, were pretreated with broussonin A or B (10 µM) for 30 min, followed by VEGF‐A (10 ng/mL) stimulation for 30 min. Briefly, cells were fixed with 3.7% paraformaldehyde, permeabilized with 0.1% Triton X‐100, blocked with 5% BSA‐PBS, and incubated with anti‐VE‐cadherin antibody. Images were observed using a Carl Zeiss Microscope (Axio Imager.M2) and AxioVision Rel. 4.8 software (Zeiss Co., Gottingen, Germany).^24^, ^27^

### 2.11 Tube formation assays

After serum starvation for 2 h, cells (4 × 10^4^ cells/mL) were plated on Matrigel^®^‐coated 24‐well plates and pretreated with broussonin A or B (1–10 µM) for 30 min, followed by VEGF‐A (10 ng/mL) for 6 h. Formation of capillary‐like structures was examined using an Olympus CKX41 inverted microscope (CAchN 10/0.25php objective) and ToupTek Toupview software (version x86, 3.5.563, Hangzhou ToupTek Photonics Co., Zhejiang, P. R. China).

### 2.12 Rat aortic ring assay

Eight‐ to nine‐week‐old male Sprague Dawley rats (250 ± 10 g) were purchased from RaonBio Inc. (Yongin, Republic of Korea). The animal experiments were conducted in accordance with the institutional guidelines. The experimental procedures were approved by the Institutional Animal Care and Use Committee at Dankook University (Cheonan, Republic of Korea). Thoracic aortic ring segments embedded in Matrigel^®^ were pretreated with broussonin A or B (10 µM) for 30 min, followed by VEGF‐A (500 ng/mL) for 3 days and then incubated with fresh broussonin A or B plus VEGF‐A every other day, and photographed on the 7^th^ day using x40 objective magnification.^28^ The area of microvessel sprouting was quantified using Adobe PhotoShop software.

### 2.13 Statistical analysis

Statistical analysis was performed using Student's *t* test and was based on at least three different experiments. The results were considered to be statistically significant when *p* < .05.

## 3 RESULTS

### 3.1 Broussonin A or B suppresses VEGF‐A‐stimulated endothelial cell proliferation

We first analysed the effect of broussonin A or B on the proliferation of HUVECs. Treatment with broussonin A or B inhibited VEGF‐A‐stimulated cell proliferation in a dose‐dependent manner and did not affect cell morphology and viability at the highest concentration used in the current study (Figure 1B,C), indicating the potential efficacy of broussonin A or B in regulating endothelial cell proliferation with little or no cytotoxicity. We next examined the effect of broussonin A or B on the cell cycle by DNA content analysis (Figure 1D). Treatment with broussonin A or B markedly inhibited VEGF‐A‐induced changes in the phase distribution of cell cycle to the levels observed in untreated controls. These findings indicate that broussonin A or B induces G_1_ cell cycle arrest, which is well correlated with suppression of cell proliferation (Figure 1B,D).^29^ Based on these findings, we examined the changes of cell cycle–related proteins in broussonin A–treated or broussonin B–treated HUVECs. As shown in Figure 1E, broussonin A markedly suppressed VEGF‐A‐induced expression of cyclin‐dependent kinases (Cdks) and cyclins to levels observed in untreated controls, resulting in pRb hypophosphorylation. In addition, broussonin B similarly inhibited pRb phosphorylation by down‐regulation of Cdk4, but not Cdk2, and cyclins. These regulatory effects of broussonin A or B on cell cycle progression and proliferation are similar to those of *B*. *kazinoki* extract in HUVECs as previously reported.^12^ Although the regulatory effect of broussonin A and B on Cdk2 expression appears slightly different, these data show the anti‐proliferative activity of broussonin A and B by blocking the G_1_‐S phase transition.

### 3.2 Broussonin A or B inhibits VEGF‐A‐stimulated endothelial cell migration, invasion and tube formation in vitro, and microvessel sprouting ex vivo

**FIGURE 2:**
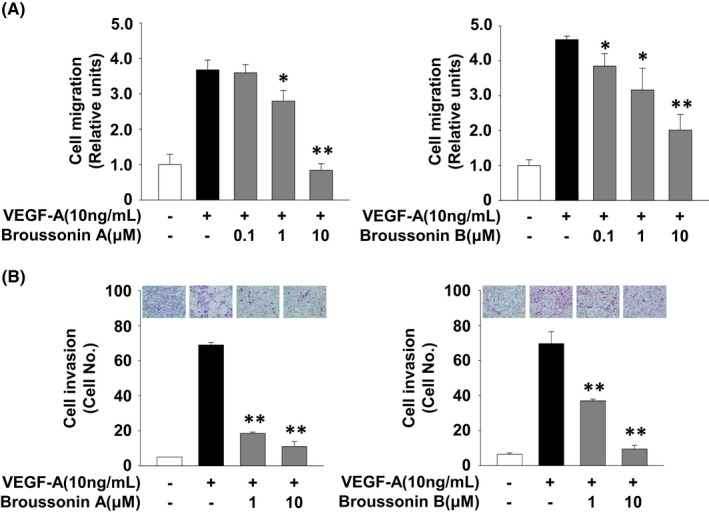
Broussonin A and B possess anti‐migratory and anti‐invasive activities in VEGF‐A‐treated HUVECs. (A) Cell migration and (B) invasion were performed as described in Materials and methods. Cells were pretreated with broussonin A or B (0.1–10 µM) for 30 min, followed by VEGF‐A (10 ng/mL) stimulation for 16 h. Results from six independent experiments (mean ± SD) were presented as (A) the fold‐increase of untreated controls or (B) the numbers of invasive cells. Statistical significance is indicated (*p < .05, **p < .01, compared with VEGF‐A‐treated cells)

**FIGURE 3:**
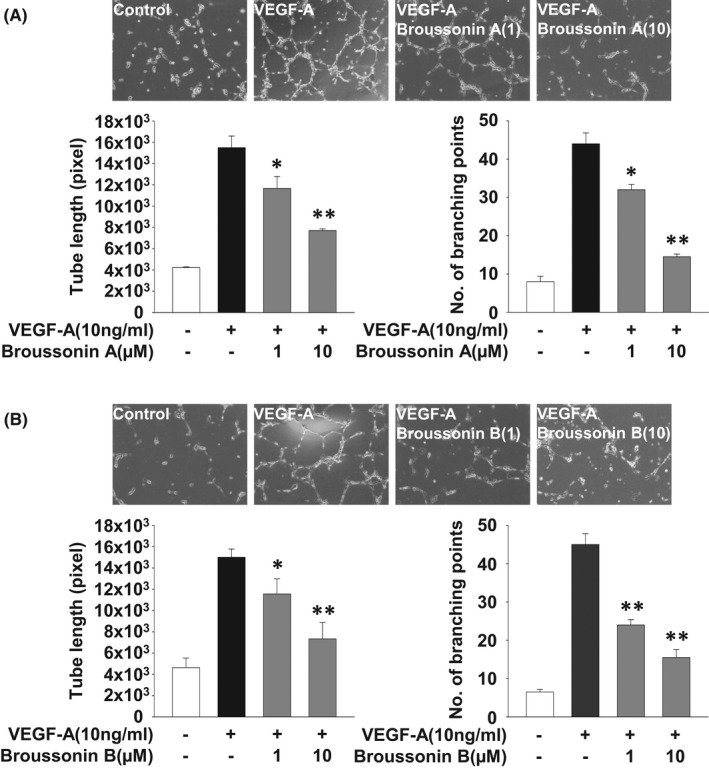
Broussonin A and B inhibit VEGF‐A‐induced capillary‐like structure formation. Tube formation assay was performed as described in Materials and methods. Cells were pretreated with broussonin A or B (0.1–10 µM) for 30 min, followed by VEGF‐A (10 ng/mL) stimulation for 6 h. Values represent the mean ± SD of at least three independent experiments. Statistical significance is indicated (*p < .05, **p < .01, compared with VEGF‐A‐treated cells)

**FIGURE 4:**
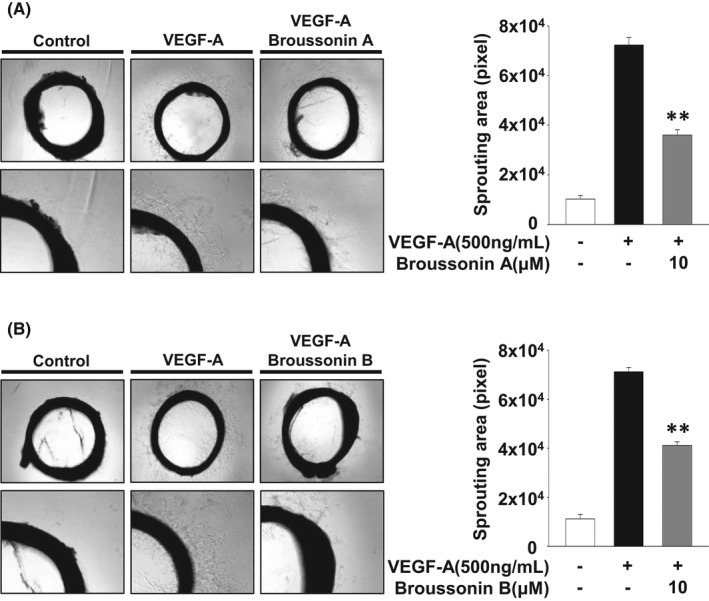
Broussonin A and B suppress VEGF‐A‐induced angiogenic sprouting ex vivo. Rat aortic ring assay was performed as described in Materials and methods. Values represent the mean ± SD of at least three independent experiments. Statistical significance is indicated (**p < .01, compared with VEGF‐A‐treated cells)

We next examined the effects of broussonin A and B on endothelial cell migration, invasion and tube formation which are essential for angiogenic responses.^30^, ^31^ Treatment with broussonin A or B dose‐dependently inhibited VEGF‐A‐stimulated cell migration and invasion (Figure 2). Moreover, both broussonin A and B significantly abrogated VEGF‐A‐induced formation of capillary‐like structures and microvessel outgrowth from rat aortic rings (Figures 3,4). Collectively, these data show the pharmacological activities of broussonin A and B in regulating VEGF‐A‐induced angiogenic responses in vitro and ex vivo.

### 3.3 Broussonin A or B regulates VEGF‐A‐induced VE‐cadherin distribution and phosphorylation

**FIGURE 5:**
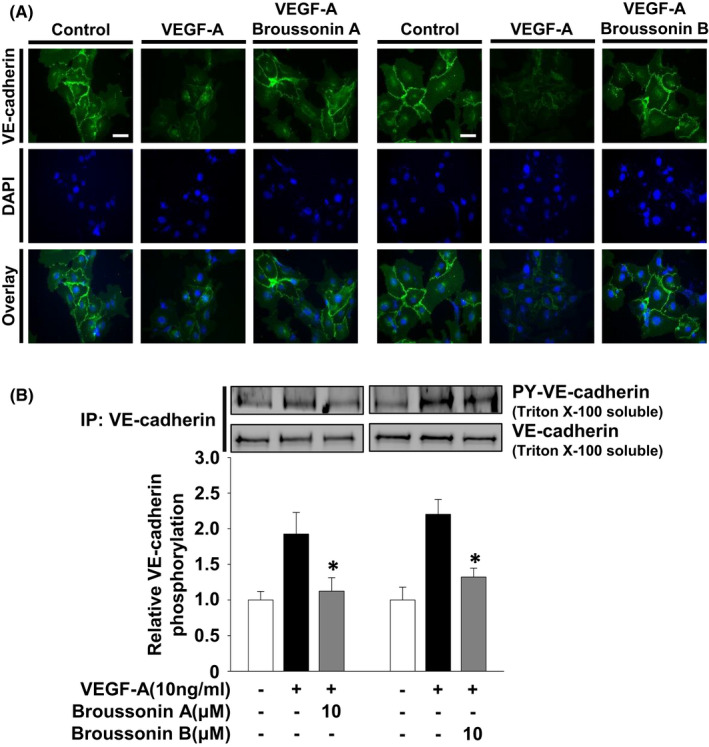
Broussonin A and B induce the localization of VE‐cadherin at cell‐cell contacts. Quiescent cells were pretreated with broussonin A or B (10 µM) for 30 min, followed by VEGF‐A (10 ng/mL) stimulation for 30 min. (A) Distribution of VE‐cadherin was determined as described in Materials and methods. DNA was stained with 4’,6‐diamidino‐2‐phenylindole (DAPI). Scale bar represents 10 µm. (B) Anti‐VE‐cadherin immunoprecipitates (IP) were Western‐blotted with anti‐phosphotyrosine or anti‐VE‐cadherin antibodies. Results shown are representative of at least three independent experiments. Integrated density values were normalized to untreated controls. Statistical significance is indicated (*p < .05, compared with VEGF‐A‐treated cells)

VEGF‐A stimulation of endothelial cells triggers the dissociation of vascular endothelial (VE)‐cadherin from β‐catenin and plakoglobin and modulates angiogenic responses such as endothelial permeability, invasion, proliferation and tube formation.^24^, ^27^, ^32^ These events can be mediated by the phosphorylation, cleavage and internalization of VE‐cadherin through VEGF‐A‐dependent signalling pathways including Src family kinases, protein tyrosine phosphatases or matrix metalloproteinases.^33^, ^34^ Therefore, we first analysed the changes in the distribution of VE‐cadherin following treatment with broussonin A and B. As anticipated, VEGF‐A stimulation dramatically induced the loss of VE cadherin at cell‐cell contacts (Figure 5A). In contrast, treatment with broussonin A or B blocked VEGF‐A‐stimulated loss of VE‐cadherin to levels observed in untreated controls. Consistent with these observations, treatment with broussonin A or B markedly inhibited VEGF‐A‐stimulated tyrosine phosphorylation of VE‐cadherin, leading to the stabilization of adherens junctions and the maintenance of endothelial barrier function (Figure 5B).^24^, ^35^ These findings suggest that inhibition of angiogenic responses by broussonin A and B is mediated at least in part through regulation of VE‐cadherin function.

### 3.4 Broussonin A or B inhibits VEGF‐A‐stimulated signalling pathways and down‐regulation of integrin β1 expression

**FIGURE 6:**
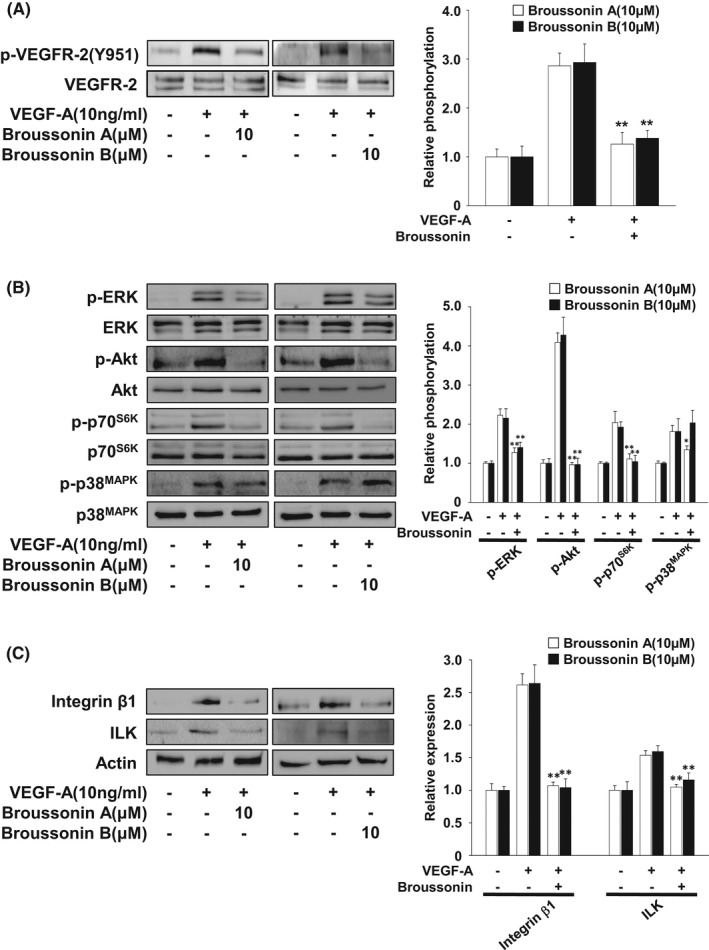
Broussonin A and B inhibit VEGF‐A‐stimulated signalling pathways and integrin β1 expression. Quiescent cells were pretreated with broussonin A or B (10 µM) for 30 min, followed by VEGF‐A (10 ng/mL) stimulation for (A) 5 min, (B) 15 min or (C) 24 h. Results shown are representative of at least three independent experiments. Integrated density values were normalized to untreated controls. Statistical significance is indicated (*p < .05, **p < .01, compared with VEGF‐A‐treated cells)

To elucidate the molecular mechanisms and targets of broussonin A and B in regulating angiogenic responses, we examined the changes in activation of VEGF‐A/VEGFR‐2 and its downstream signalling pathways including p70 S6 kinase (p70^S6K^), Akt, extracellular signal‐regulated kinase (ERK) and p38 mitogen‐activated protein kinase (p38^MAPK^).^36^, ^37^ As anticipated, VEGF‐A markedly induced the phosphorylation of VEGFR‐2, p70^S6K^, Akt, ERK and p38^MAPK^, as compared with untreated controls (Figure 6A,B). Treatment with broussonin A or B significantly inhibited the phosphorylation of VEGFR‐2 on Tyr 951 residue, one of the major phosphorylation sites in VEGFR‐2, which is associated with the regulation of endothelial adherens junctions through VE‐cadherin phosphorylation (Figure 5B).^38^ Broussonin A inhibited VEGF‐A‐stimulated phosphorylation of p70^S6K^, Akt, ERK and p38^MAPK^ (Figure 6B). In contrast, broussonin B did not affect the phosphorylation of p38^MAPK^ in response to VEGF‐A stimulation.

Furthermore, treatment with broussonin A or B markedly suppressed VEGF‐A‐induced expression of integrin β1 and integrin‐linked kinase (ILK), a key kinase in integrin downstream signalling pathways, which are closely associated with angiogenesis and tumour progression (Figure 6C).^39^, ^40^, ^41^ These regulatory roles and mechanisms of broussonin A and B in VEGF‐A/VEGFR‐2‐mediated signalling networks are similar to those of *B*. *kazinoki* extract,^12^ suggesting that broussonin A and B can be the pharmacologically effective constituents from *B*. *kazinoki* in regulating angiogenic responses.

### 3.5 Broussonin A or B inhibits proliferation and invasion of cancer cells

**FIGURE 7:**
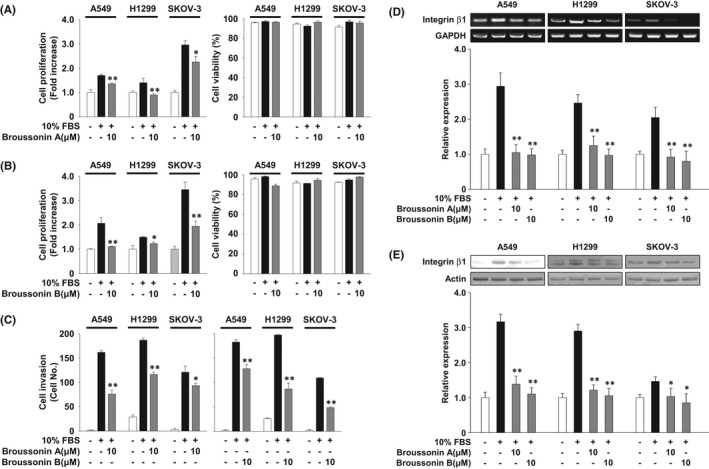
Broussonin A and B have anti‐tumour activity against A549, H1299 and SKOV‐3 cells. Quiescent cells were pretreated with broussonin A or B (10 µM) for 30 min, followed by 10% FBS stimulation for (A, B, D, E) 24 h or (C) 16 h. The results from at least three independent experiments (mean ± SD) are presented as (A, B; left panel) the fold‐increase of untreated controls, (A, B; right panel) the percentage of viable cells of total cell counts or (C) the numbers of invasive cells. (D) RT‐PCR and (E) Western blot analyses were performed as described in Materials and methods. Results shown are representative of at least three independent experiments. Integrated density values were normalized to untreated controls. Statistical significance is indicated (*p < .05, **p < .01, compared with 10% FBS‐treated cells)

Based on inhibitory effects of broussonin A and B on angiogenic responses, we next examined the ability of broussonin A and B to regulate proliferation and invasion in NSCLC p53 wild‐type A549 and p53‐deficient H1299 cells as well as ovarian cancer p53‐deficient SKOV‐3 cells (Figure 7). Treatment of broussonin A or B inhibited mitogen‐stimulated proliferation of NSCLC and ovarian cancer cells. H1299 cells were more sensitive to broussonin A–mediated inhibition of cell proliferation, as compared with A549 or SKOV‐3 cells (Figure 7A). In contrast, broussonin B–mediated inhibition of proliferation in A549 cells was found to be more potent than that in H1299 or SKOV‐3 cells (Figure 7B). As shown in Figure 7C, treatment with broussonin A or B significantly inhibited mitogen‐stimulated cell invasion. Unlike the inhibitory patterns of cell proliferation, broussonin A–mediated inhibition of cell invasion appeared to be more potent in A549 and H1299 cells than in SKOV‐3 cells. In addition, broussonin B shows stronger inhibitory activity against H1299 and SKOV‐3 than A549 cells, indicating that broussonin B–mediated inhibition of cell invasion might be dependent on p53 protein levels. Finally, treatment with broussonin A or B markedly suppressed mitogen‐induced expression of integrin β1 in NSCLC and ovarian cancer cells (Figure 7D,E; Supplementary material). Although the inhibitory potency of broussonin A and B in regulating cancer cell proliferation and invasion appears to be dependent on the specific cell/tissue types or p53 expression status, these findings suggest anti‐tumour activities of broussonin A and B in NSCLC and ovarian cancer cells might be correlated with suppression of integrin β1 expression.

## 4 DISCUSSION

In the current study, we report that both broussonin A and B negatively regulate VEGF‐A‐induced in vitro endothelial cell responses including proliferation, migration, invasion and capillary‐like structure formation as well as ex vivo angiogenesis. The mechanism of these anti‐angiogenic effects involves inactivation of VEGF‐A/VEGFR‐2 downstream signalling pathways such as ERK, Akt, p70^S6K^ and p38^MAPK^, redistribution of VE‐cadherin to cell‐cell contacts, and down‐regulation of integrin β1 and ILK. In addition, both broussonin A and B exert anti‐proliferative and anti‐invasive activities against NSCLC and ovarian cancer cells. Our findings demonstrate the pharmacological potential of broussonin A and B in the regulation of pathological angiogenic responses associated with cancer growth and progression.

Integrins, transmembrane receptors that facilitate cell‐extracellular matrix and cell‐cell interactions, mediate a wide range of cellular responses including adhesion, migration, proliferation, invasion and angiogenesis associated with tumour growth and progression.^4^, ^39^, ^42^, ^43^ These cellular responses are mediated by cross‐talk between integrins and receptor tyrosine kinases including VEGFR‐2, platelet‐derived growth factor receptor and epidermal growth factor receptor.^40^, ^41^, ^44^, ^45^ Previous studies demonstrate that integrin β1 interacts with VEGFRs or neuropilin‐1, a co‐receptor for VEGF‐A, and modulates adhesion, invasion, proliferation, survival and angiogenesis in various types of cells, suggesting that selective inhibition of integrin β1 function as well as VEGF/VEGFR signalling pathways might be a potential therapeutic strategy for the treatment of angiogenesis‐related disorders.^46^, ^47^, ^48^, ^49^, ^50^ Strategies to modulate integrin function involve blockade of ligand binding, inhibition of integrin downstream signalling pathways, or regulation of integrin expression.^43^ Although the effects of broussonin A and B on integrin functions including ligand binding and signalling networks remain to be further determined, treatment with broussonin A or B markedly suppresses VEGF‐A‐ and mitogen‐induced expression of integrin β1 in endothelial cells and cancer cells, respectively.

Cell adhesion, migration and invasion are tightly controlled by the changes in the expression of adhesion molecules such as integrins and cadherins and/or activity of matrix metalloproteinases.^51^, ^52^ Treatment with broussonin A or B markedly prevented VEGF‐A‐induced VE‐cadherin tyrosine phosphorylation and the loss of VE‐cadherin at cell‐cell contacts. These findings are well correlated with inhibition of VEGFR‐2 phosphorylation on Tyr 951 residue as previously reported.^38^, ^53^ Broussonin A‐ and B‐mediated regulation of VE‐cadherin distribution might inhibit the migratory and invasive potential of endothelial cells in response to VEGF‐A stimulation. Collectively, we demonstrate here that both broussonin A and B exert anti‐angiogenic activity through inactivation of VEGF‐A/VEGFR‐2 signalling pathways and regulation of cell adhesion molecules such as VE‐cadherin and integrin β1.

In addition to anti‐angiogenic activity, both broussonin A and B have anti‐proliferative and anti‐invasive activities against NSCLC and ovarian cancer cells concomitant with suppression of integrin β1 expression, independently of p53 levels. In conclusion, our results provide significant insights into the regulatory roles and therapeutic efficacy of broussonin A and B in angiogenesis and cancer progression and warrant preclinical evaluation and development as a promising therapeutic agent for the treatment of a wide range of angiogenesis‐related diseases including cancer.

## CONFLICT OF INTEREST

The authors declare that there are no conflicts of interest.

## AUTHOR CONTRIBUTIONS

**Jae Hyeon Kim:** Conceptualization (equal); Investigation (lead); Methodology (lead); Validation (equal); Visualization (equal); Writing – original draft (equal); Writing – review & editing (equal). **Sunho Kim:** Data curation (equal); Formal analysis (equal); Investigation (equal). **Surim Han:** Data curation (equal); Formal analysis (equal); Investigation (equal). **Eun‐Kyung Ahn:** Conceptualization (supporting); Data curation (equal); Investigation (supporting). **Young‐Rak Cho:** Conceptualization (supporting); Data curation (equal); Investigation (supporting). **Wonsik Jeong:** Data curation (equal); Investigation (supporting). **Sung Joon Kim:** Data curation (supporting); Investigation (supporting). **Gyu‐Un Bae:** Conceptualization (supporting); Resources (supporting). **Joa Sub Oh:** Conceptualization (supporting); Resources (supporting). **Dong‐Wan Seo:** Conceptualization (lead); Data curation (equal); Funding acquisition (lead); Resources (lead); Supervision (lead); Writing – original draft (lead); Writing – review & editing (lead).

## Supporting information

Supplementary Material

## Data Availability

The data that support the findings of this study are available from the corresponding author upon reasonable request.
