# Supplementary material for: Broussonin A– and B–mediated inhibition of angiogenesis by blockade of VEGFR‐2 signalling pathways and integrin β1 expression
Source: J Cell Mol Med. 2022 Jan 6;26(4):1194–205. doi: 10.1111/jcmm.17173 (PMC8831976; doi:10.1111/jcmm.17173)
Supplement: Supplementary file 1 — Supplementary Material [file JCMM-26-1194-s001.pptx]

## Slide 1
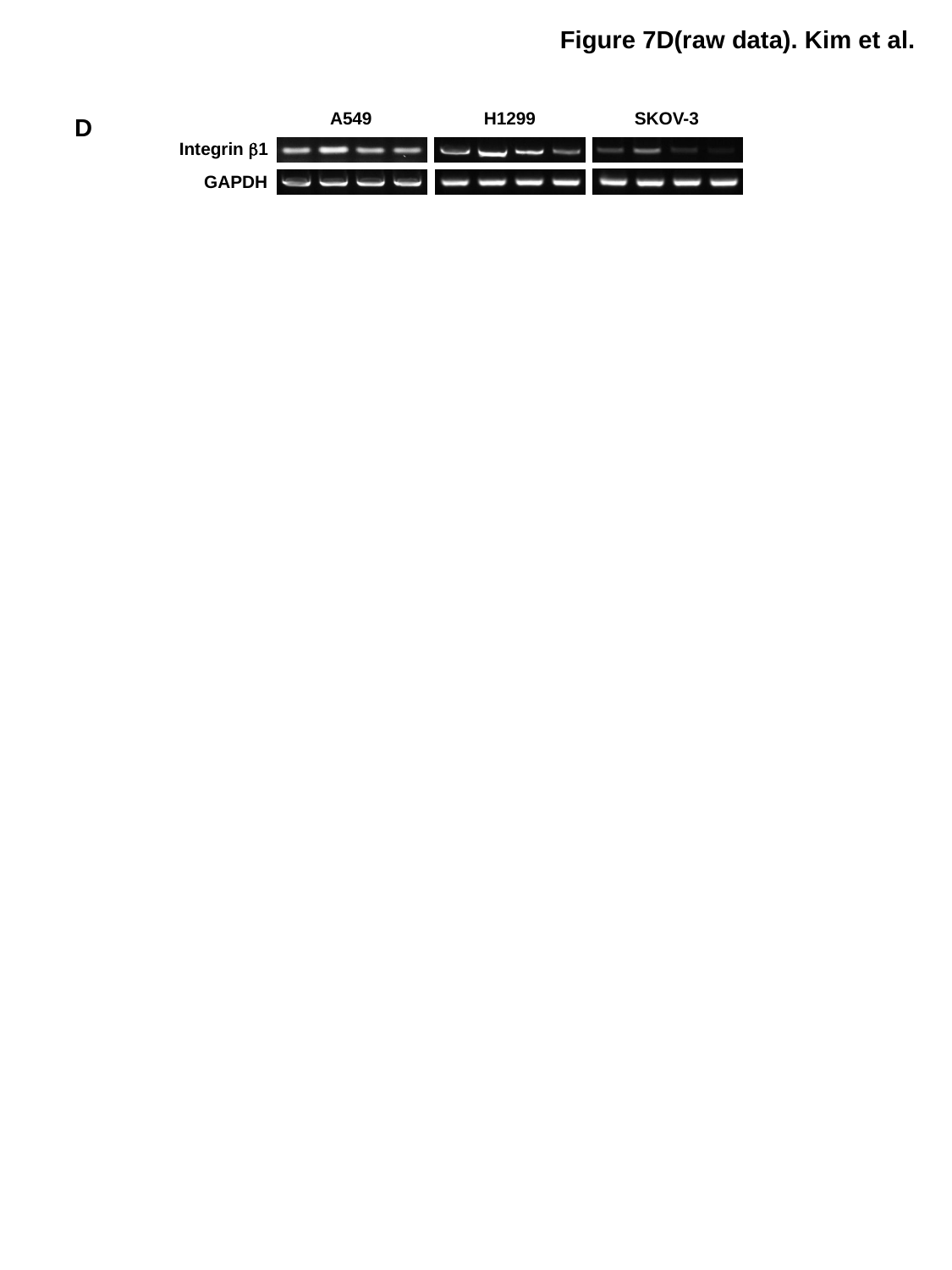

Figure 7D(raw data). Kim et al.
H1299
SKOV-3
A549
Integrin b1
GAPDH
D

## Slide 2
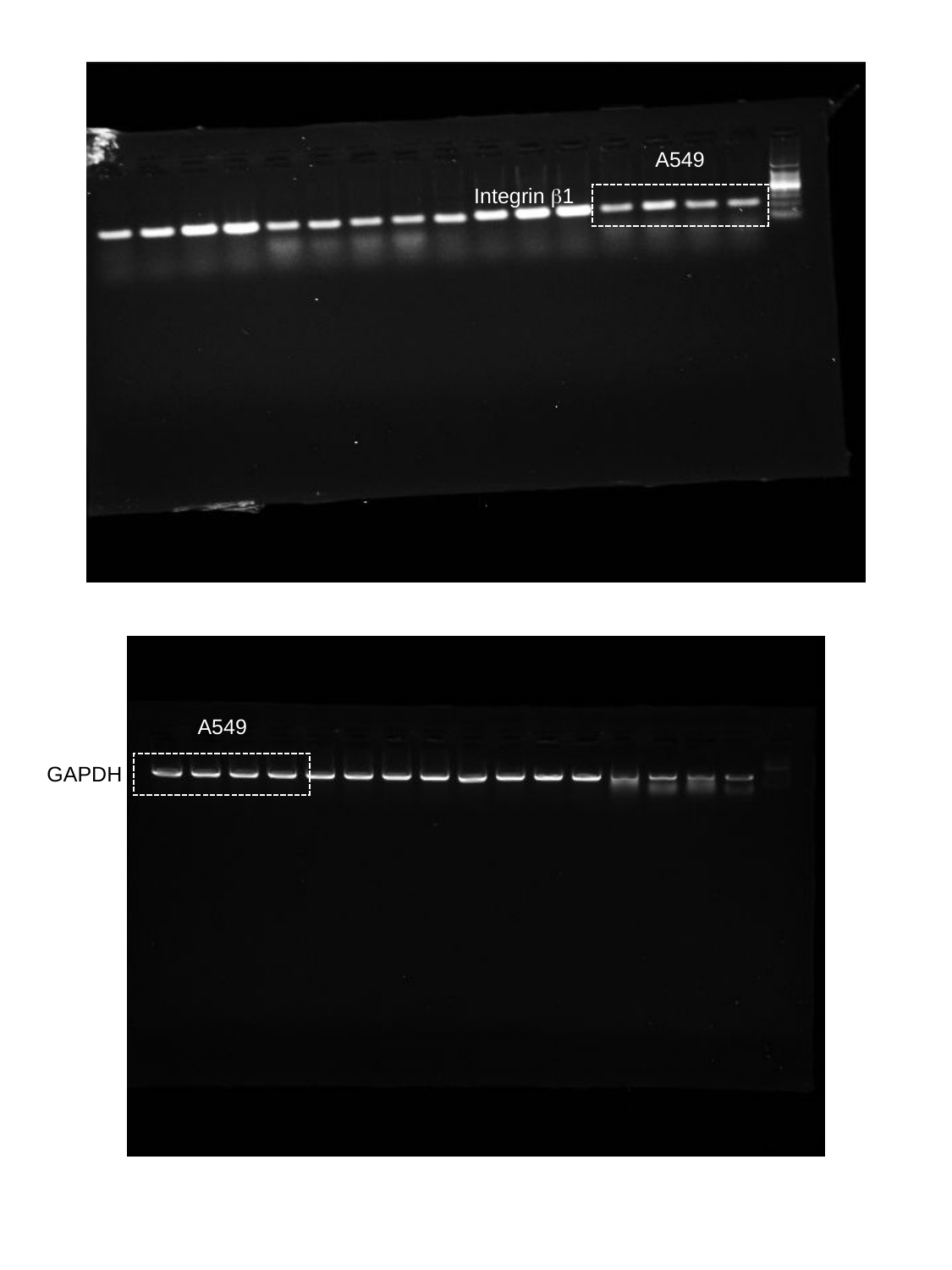

A549
Integrin b1
A549
GAPDH

## Slide 3
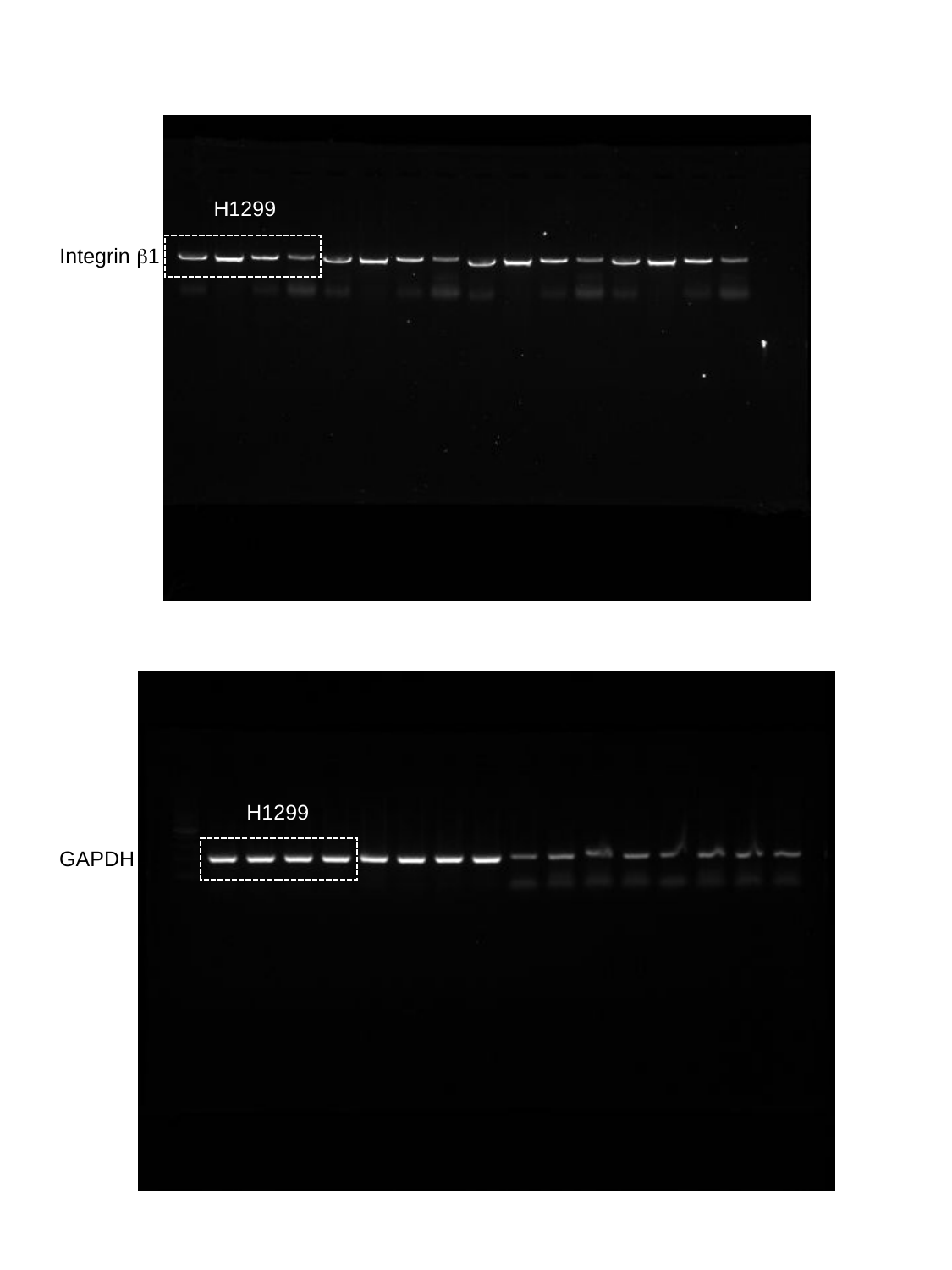

H1299
Integrin b1
H1299
GAPDH

## Slide 4
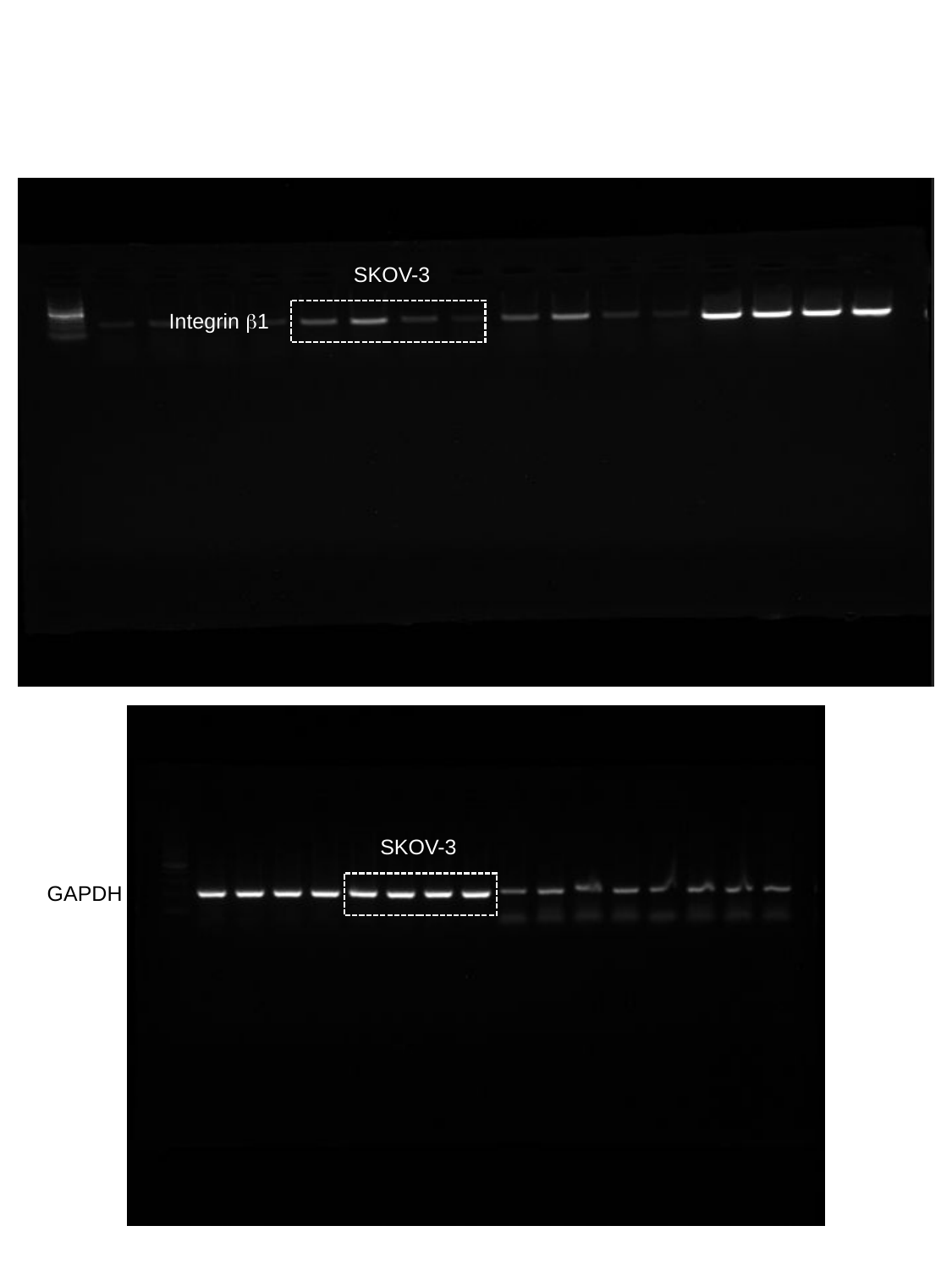

SKOV-3
Integrin b1
SKOV-3
GAPDH

## Slide 5
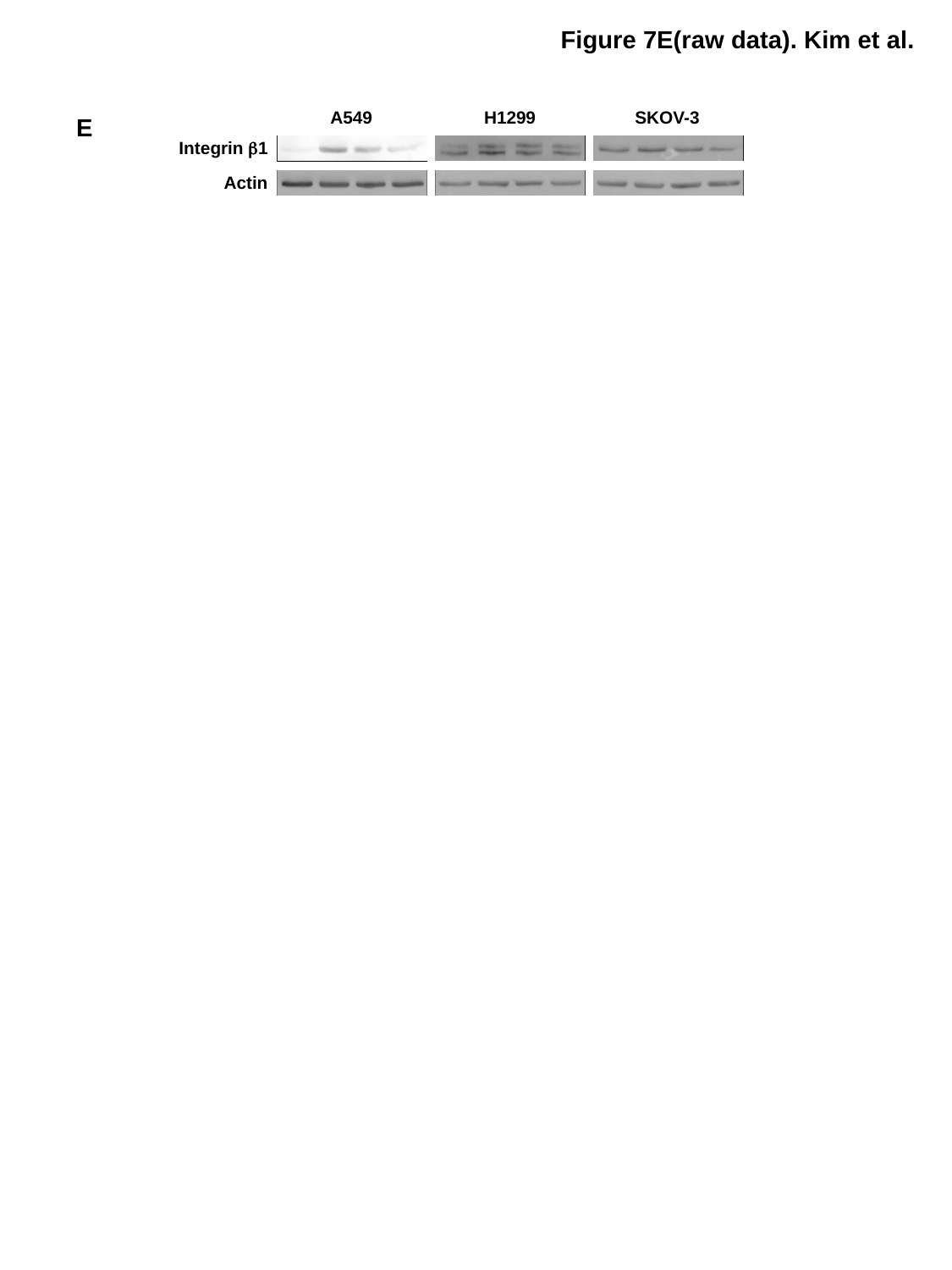

Figure 7E(raw data). Kim et al.
H1299
SKOV-3
A549
Integrin b1
Actin
E

## Slide 6
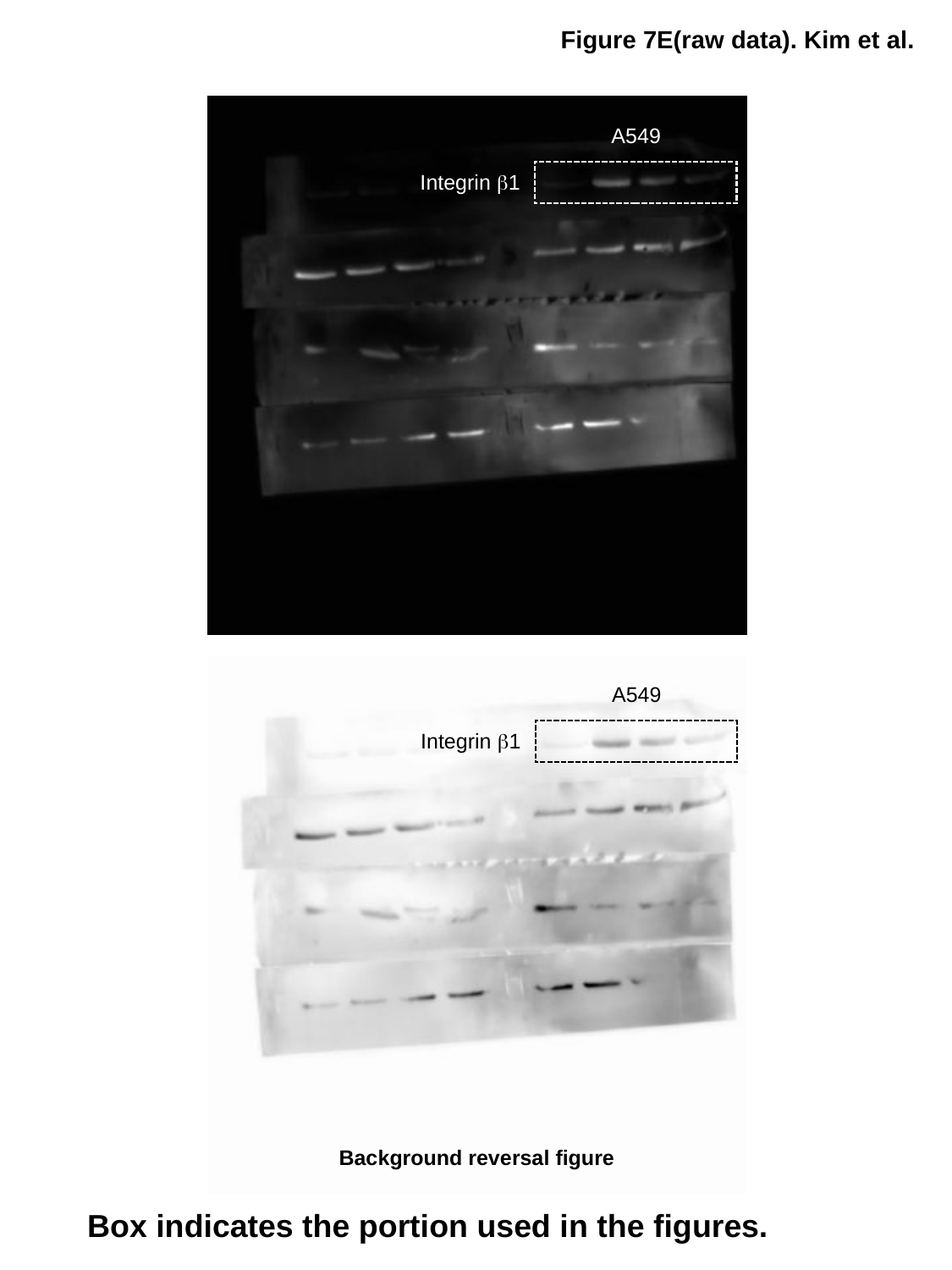

Figure 7E(raw data). Kim et al.
A549
Integrin b1
A549
Integrin b1
Background reversal figure
Box indicates the portion used in the figures.

## Slide 7
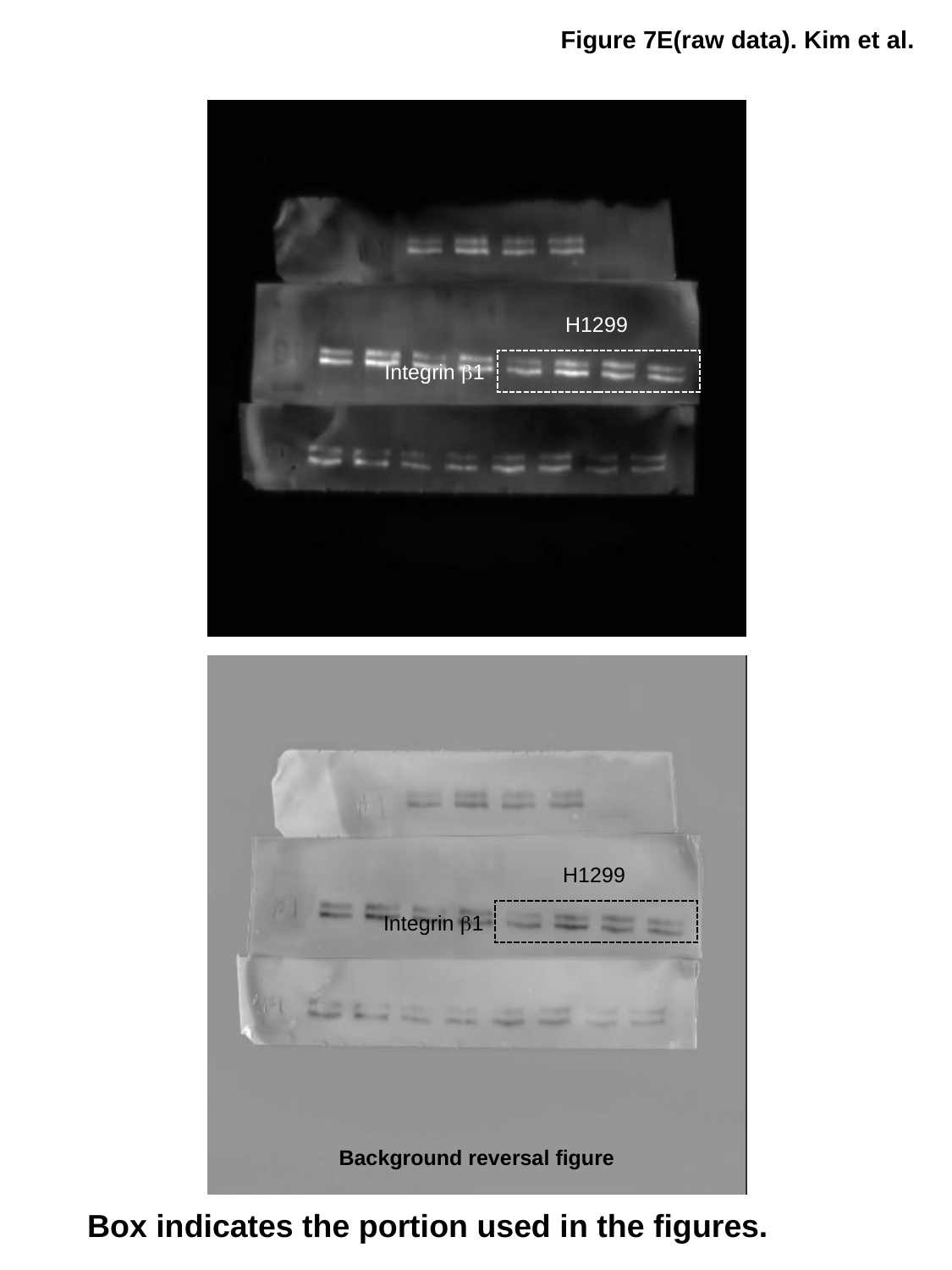

Figure 7E(raw data). Kim et al.
H1299
Integrin b1
H1299
Integrin b1
Background reversal figure
Box indicates the portion used in the figures.

## Slide 8
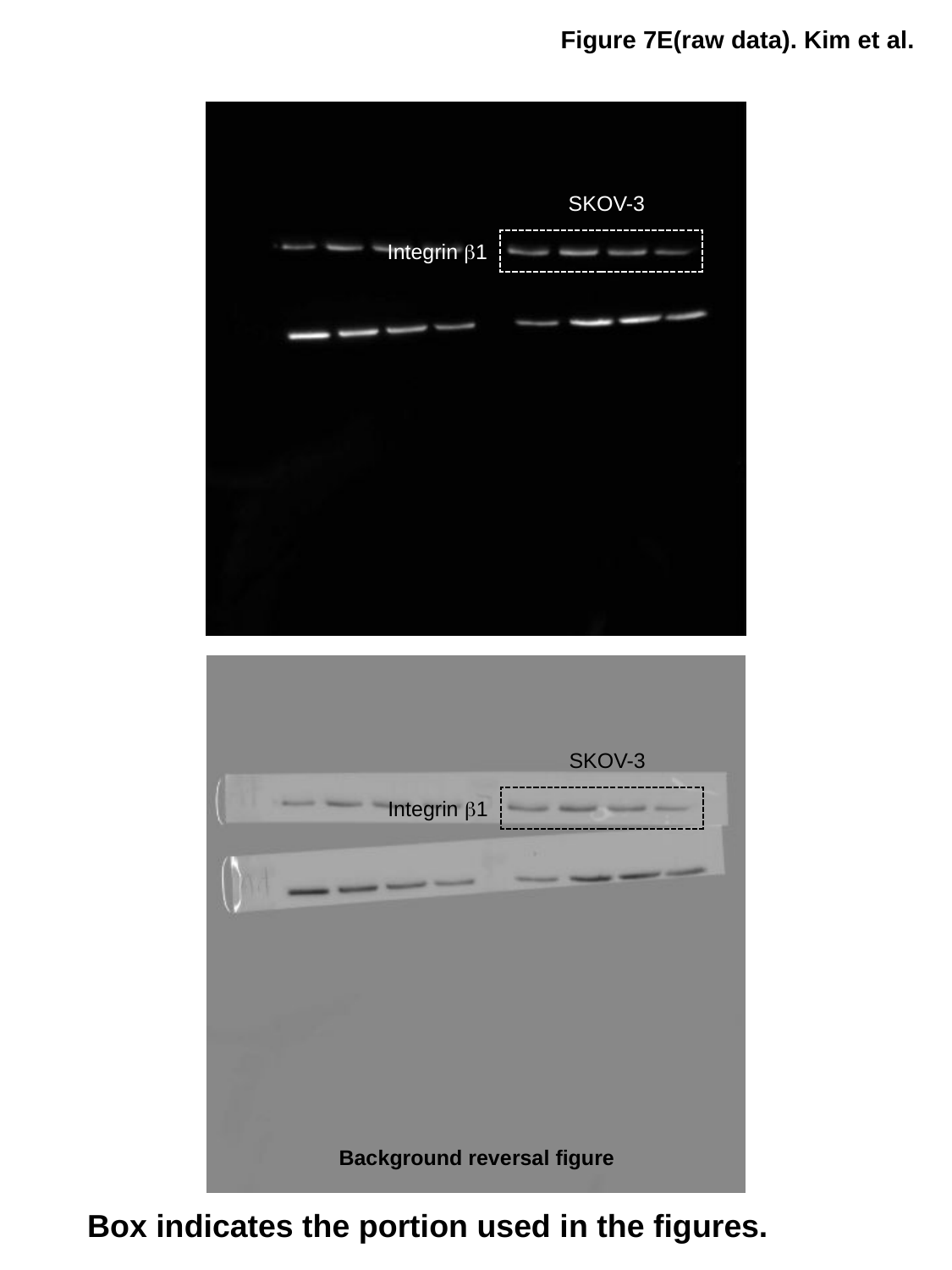

Figure 7E(raw data). Kim et al.
SKOV-3
Integrin b1
SKOV-3
Integrin b1
Background reversal figure
Box indicates the portion used in the figures.

## Slide 9
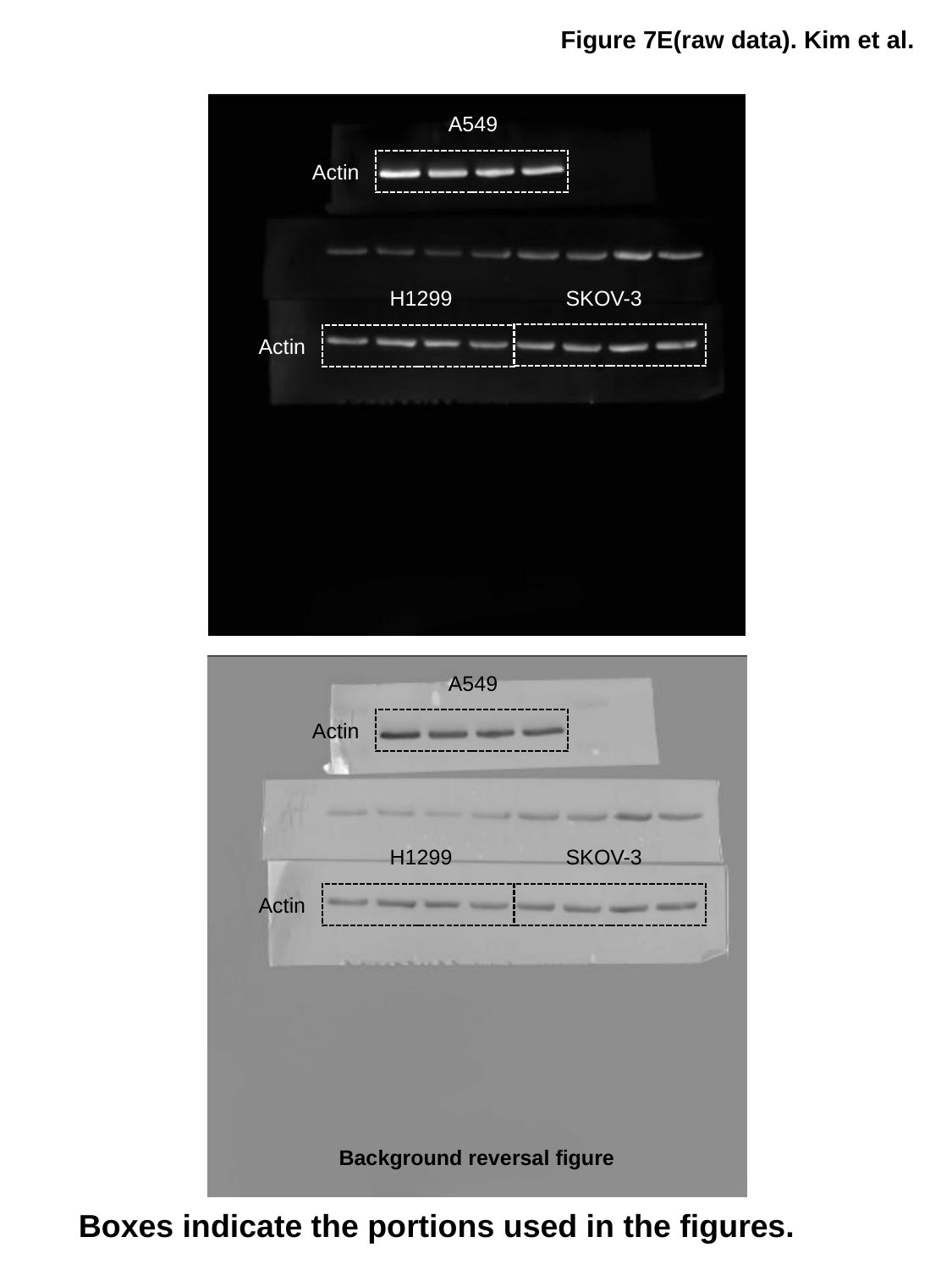

Figure 7E(raw data). Kim et al.
A549
Actin
SKOV-3
H1299
Actin
A549
Actin
SKOV-3
H1299
Actin
Background reversal figure
Boxes indicate the portions used in the figures.
